# Effects of Lifestyle Modification Programs on Cardiac Risk Factors

**DOI:** 10.1371/journal.pone.0114772

**Published:** 2014-12-09

**Authors:** Moaven Razavi, Stephen Fournier, Donald S. Shepard, Grant Ritter, Gail K. Strickler, William B. Stason

**Affiliations:** Schneider Institutes for Health Policy, Heller School, Brandeis University, Waltham, Massachusetts, 02454–9110, United States of America; Children’s National Medical Center, Washington, United States of America

## Abstract

Medicare conducted a payment demonstration to evaluate the effectiveness of two intensive lifestyle modification programs in patients with symptomatic coronary artery disease: the Dr. Dean Ornish Program for Reversing Heart Disease (Ornish) and Cardiac Wellness Program of the Benson-Henry Mind Body Institute. This report describes the changes in cardiac risk factors achieved by each program during the active intervention year and subsequent year of follow-up. The demonstration enrolled 580 participants who had had an acute myocardial infarction, had undergone coronary artery bypass graft surgery or percutaneous coronary intervention within 12 months, or had documented stable angina pectoris. Of these, 98% completed the intense 3-month intervention, 71% the 12-month intervention, and 56% an additional follow-up year. Most cardiac risk factors improved significantly during the intense intervention period in both programs. Favorable changes in cardiac risk factors and functional cardiac capacity were maintained or improved further at 12 and 24 months in participants with active follow-up. Multivariable regressions found that risk-factor improvements were positively associated with abnormal baseline values, Ornish program participation for body mass index and systolic blood pressure, and with coronary artery bypass graft surgery. Expressed levels of motivation to lose weight and maintain weight loss were significant independent predictors of sustained weight loss (p = 0.006). Both lifestyle modification programs achieved well-sustained reductions in cardiac risk factors.

## Introduction

Cardiovascular disease is a major cause of mortality and disability despite widespread efforts to control cardiac risk factors through diet, exercise, and medications. Though death rates due to cardiovascular disease (CVD) declined 30.6% between 1998 and 2008, in 2008, 82.6 million U.S. citizens had CVD and 811,900 died from it, its associated direct and indirect costs totaled $297.7 billion. In 2009, 416,000 underwent coronary artery bypass graft (CABG) surgery and 596,000 received percutaneous coronary interventions (PCIs) [1].

Cardiac risk factors contribute importantly to the development of coronary heart disease (CHD). Obesity, diabetes, serum cholesterol levels, hypertension, smoking, inadequate exercise, and stress each provides an important target for efforts to reduce associated morbidity and mortality. Cardiac rehabilitation (CR) programs have been the most thoroughly studied. These programs typically begin in the hospital setting following a cardiac event and include monitored aerobic exercise, dietary advice, and other services to achieve lipid control, weight loss, and stress modification. [2], [3], [4], [5], [6], [7] Meta-analyses of controlled trials of CR have demonstrated 15% to 28% reductions in all-cause mortality. [8], [9], [10], [11], [12] Generalizability of these findings is limited, however, because most studies included relatively few older persons, women, members of racial/ethnic minorities, or high-risk patients including those with congestive heart failure.

Programs aimed at secondary cardiac prevention have also been conducted in health care centers outside of hospitals or at home. A meta-analysis that included 63 randomized trials of this type and over 21,000 patients with CHD showed a 15% reduction in all-cause mortality and a 17% reduction of acute myocardial infarctions (AMIs). [8], [12] Effects were similar whether or not the risk factor reduction program included structured exercise. More than half of the studies reported favorable effects on cardiac risk factors including cholesterol profiles and functional status, though effect sizes were generally small and were statistically significant in only half of studies. Ornish reported that intensive lifestyle changes may reverse coronary artery arteriosclerosis [13], [14].

## Methods

Medicare conducted a payment demonstration from 2000 to 2008 to examine the effects of intense lifestyle modification programs in patients with symptomatic coronary artery disease or recent cardiac events on cardiac risk factors and the progression of CVD, health outcomes, and the cost-effectiveness of care. The demonstration involved two multisite programs: The Dr. Dean Ornish Program for Reversing Heart Disease (Ornish) and the Cardiac Wellness Program of the Benson-Henry Mind/Body Medical Institute (MBMI). Enrollment lasted from 2000 until 2006 and aimed to achieve two years of follow-up in each participant. This report focuses on changes in cardiac risk factors that were achieved over the two-year follow-up period.

### Demonstration Design

The demonstration involved 12 Ornish sites and 5 MBMI sites and included both fee-for-service and managed care Medicare beneficiaries. Enrollment began in 2000 for the Ornish program and in 2002 for the MBMI program and ended for both programs in February 2006. Two years of follow-up for all enrollees was completed in February 2008. Objectives were to reduce the levels of cardiac risk factors and the risk of future cardiovascular events in participants. Each program included an intense three-month intervention period followed by nine months of less frequent sessions and greater emphasis on home maintenance of healthy lifestyle behaviors. Follow-up continued for an additional 12 months.

### Evaluation Design

This report focuses on enrollment into the two programs and their effects on cardiac risk factors, exercise tolerance, and the occurrence of cardiovascular events during two years of follow-up. During the demonstration, Medicare reimbursed each 12-month program’s negotiated fees that were linked to active participation during each 3-month period.

### Eligibility Criteria

Participants were 65 years of age or older, had had an AMI, CABG, or PCI within the previous 12 months, or had stable angina pectoris with cardiac ischemia documented by coronary angiography or a stress echocardiogram. Patients with high-risk cardiac conditions were excluded, including those with a 50 percent or greater narrowing of the left main coronary artery, three-vessel disease, a left ventricular ejection fraction of less than 30%, two-vessel disease with occlusions of 70% or more and an ejection fraction of less than 30%, unstable angina, high-risk exercise or nuclear stress test results, or American Heart Association Level IV congestive heart failure. Individuals with impaired cognitive function were also excluded, as were those whose travel time from home to the program site was more than 90 minutes under usual traffic conditions.

### Lifestyle Modification Program Interventions

Each program included exercise, nutrition counseling, stress management, and small group support. The Ornish program had a 12-week intense phase that included three 4-hour sessions in week 1, two in weeks 2 through 11, and three in week 12. Nutrition counseling targeted a vegetarian diet with 10% or fewer calories from fat. During the remainder of the first year, participants received either 12 or 24 weeks of two-hour weekly sessions or 40 weeks of four-hour weekly sessions based on their medical risk stratification and adherence to lifestyle change guidelines. In the second year, they were offered assistance in obtaining self-directed community follow-up and were periodically reevaluated.

The MBMI program included similar components but was somewhat less intense. Participants received one 3-hour session per week during the first 13 weeks. Emphasis was placed on adherence to the American Heart Association diet (30% or fewer calories from fat), group support and behavior change, and one-on-one health contracting and assessment sessions. During the rest of the first year, they attended 3-hour sessions twice a month. At the end of the year, they were given a program completion certificate, revised health contract, and information about “graduate” groups and community resources. Outcomes were monitored during the second year.

### Data Sources

The clinical sites provided baseline data on candidates to the Delmarva Foundation, Inc., a Professional Review Organization in Maryland that had contracted with the Centers for Medicare and Medicaid Services (CMS) to determine beneficiary eligibility for the demonstration and to monitor program implementation. During programs, monthly data were provided to Delmarva to document changes in cardiac risk factors and psychological outcomes, adherence to program protocols, changes in medication regimens, and any adverse clinical events that had occurred. Each participant was asked to complete a mailed survey at the time of enrollment to provide information on sociodemographic characteristics, prior health-related behaviors, and psychosocial characteristics. Monitored cardiac risk factors included body weight, systolic and diastolic blood pressure, total serum cholesterol, low density and high density cholesterol sub-fractions, triglycerides, and hemoglobin A1c in patients with documented diabetes.

### Statistical Analysis

Descriptive statistics compare the baseline characteristics of participants in the Ornish and MBMI programs and document risk factor changes at the end of the intense intervention period (3 months), end of the active program (12 months), and end of the follow-up period (24 months). Analysis of the program’s effects on cardiac risk factors required that values be available at both baseline and the end of each follow-up period. Targeted risk factors include low density lipoprotein (LDL), body-mass index (BMI), systolic blood pressure (SBP), diastolic blood pressure (DBP), and high density lipoproteins (HDL). Changes were calculated from baseline both for participants with any data at a specified time point (denoted by ANY) and those with full attendance (i.e., provided data at each time point, denoted as FULL). The analysis with ANY data provides the largest and most representative sample, while the FULL data analysis provides a consistent cohort for comparison across time periods. Analysis of responses to a baseline participant survey focused on race, level of education, smoking history, prior efforts to lose weight and maintain weight loss, support from family and friends, the ability to “take charge” of life’s challenges, and an overall rating of current health status.

To adjust for differences among participants in comparing the programs, multivariate regression analyses of changes in each cardiac risk factor adjusted for the baseline level of the risk factor, type of program (MBMI or Ornish), demonstration time period (measured in quarter years, in both linear and quadratic terms), qualifying cardiac diagnosis, and the age, gender, race, and educational level of the participant. The inclusion of the quadratic term for time period allowed estimating how program impacts varied over the follow-up period.

### IRB Approval

This evaluation was approved by Brandeis University’s Institutional Review Board (IRB). All participants provided written informed consent.

## Results

### Participation in a Lifestyle Program

Enrollment and program completion rates are summarized in Table 1. A total of 580 Medicare beneficiaries participated in the demonstration, consisting of 440 in the MBMI program and 140 in the Ornish program. Of these, 98% completed the intense 3-month intervention, 71% the 12-month intervention, and 56% the subsequent year of follow-up. Completion rates were similar in the two programs during the active program year but were higher in the MBMI program at the end of the follow-up year (58% versus 47%). Reasons for disenrollment from the active program included medical causes in 63 participants, non-compliance in 64, personal reasons in 44, and closures of clinical sites in 38. Less frequent reasons included family issues, relocations, commuting problems, and conflicts with work. Fourteen participants left the program to undergo surgery, including five who received cardiac revascularization procedures. Eight patients died during the demonstration period, including two from cardiac causes. Neither cardiac death occurred during the active intervention period.

**Table 1:** Completion Rates for Lifestyle Modification Programs.

Target Time(months)	Time IntervalAccepted (months)	Ornish Program(n = 140)	MBMI Program(n = 440)	Programs Combined(n = 580)
Enrollment	0	100%	100%	100%
3	1–6	98%	98%	98%
12	7–18	67%	72%	71%
24	19–30	47%	58%	56%

MBMI denotes the Cardiac Wellness Program of the Benson-Henry Mind Body Institute. Ornish denotes The Dean Ornish Program for Reversing Heart Disease. The observation closest to the target time was taken, provided it was within the stated interval.

### Baseline Characteristics of Participants

The mean age of participants was 71.5 years, 65% were male, and 85% were white. (Table 2) They were highly educated and included 60% with at least some college education and 38% with a 4-year college degree or more. The frequencies of qualifying cardiac events differed between the Ornish and MBMI programs (chi square p<0.05). More participants in the Ornish program had stable angina (19% vs. 14%) or had received PCIs (41% vs. 31%), while more in the MBMI program had undergone CABG surgery (26% vs. 15%). Intervals between the onset of the qualifying clinical event and enrollment into the program were three months longer in the Ornish program than the MBMI program (270 days vs. 169 days, p = 0.02) due in part to the fact that enrollment was by cohort into the Ornish program and continuous into the MBMI program. Large majorities of participants were receiving antilipemics (87%) and/or beta-blockers (79%) at the time of enrollment.

**Table 2:** Baseline Characteristics of Participants in the Medicare Lifestyle Modification Demonstration.

Characteristic	All Participants(N = 580)	Ornish Program(N = 140)	MBMI Program(N = 440)	p value ofdiff
Age (mean years)	71.5	71.2	71.6	N
Gender (% male)	65%	66%	65%	N
Race (% white)	85%	86%	85%	N
Education				
Less than HS	8%	5%	10%	N
HS graduate or GED	33%	37%	32%	N
Some college	22%	20%	22%	N
4-year college degree	15%	17%	14%	N
Some post-graduate credits	23%	21%	23%	N
Type of Qualifying Event				
AMI, no cardiac procedure	28%	25%	29%	N
Angina only	15%	19%	14%	N
CABG	23%	15%	26%	0.008
PCI only	33%	41%	31%	0.032
Medications at Baseline				
Antilipemics (%)	87%	81%	88%	0.017
Beta-blockers (%)	79%	79%	80%	N

MBMI is the Benson-Henry Mind/Body Medical Institute. Ornish is The Dean Ornish Program for Reversing Heart Disease. AMI is acute myocardial infarction; CABG is coronary artery bypass graft surgery; PCI is percutaneous coronary intervention. Levels of statistical significance between programs are based on 2×2 chi-squares for each level of categorical variables and t-tests for continuous variables. N is not significant.

Baseline levels of cardiac risk factors are summarized in Table 3. The average participant in both programs combined was moderately overweight with a mean body mass index (BMI) of 28.8 kg/m^2^, had normal total cholesterol (161 mg/dl) and LDL cholesterol (89 mg/dl), had mean SBP of 133 mmHg, and mean DBP of 73 mmHg. These baseline risk factor levels reflect, in part, the high proportions of participants who were receiving antilipemics, beta-blockers, and/or antihypertensive agents when they entered the demonstration. The mean baseline systolic blood pressure was higher in MBMI than Ornish program participants (134.6 mmHg vs. 128.8 mmHg; p = 0.002), and baseline cardiac functional capacity was lower in MBMI program participants (6.9 METs vs. 8.2 METs; p<0.001).

**Table 3:** Baseline Levels of Cardiac Risk Factors.

	OrnishProgram		MBMIProgram		Differences	
Risk Factor	Mean	SD	Mean	SD	Mean	p-value
Body weight (lbs.)	184.0	36.1	183.4	34.2	0.6	0.864
BMI (kg/m3)	28.8	4.4	28.9	4.7	−0.1	0.870
SBP (mmHg)	128.8	17.1	134.6	19.0	−5.7	0.002
DBP (mmHg)	72.4	10.1	73.0	10.5	−0.5	0.613
Total cholesterol (mg/dl)	163.7	47.5	160.3	36.2	3.4	0.367
LDL (mg/dl)	89.6	39.3	89.2	29.4	0.4	0.905
HDL (mg/dl)	44.9	12.8	43.6	12.2	1.2	0.312
Triglycerides (mg/dl)	145.5	77.3	139.9	75.4	5.7	0.044
Cardiac functional capacity (METs)	8.2	2.1	6.9	2.0	1.3	0.000

SD is standard deviation; BMI is body mass index; SBP is systolic blood pressure; DBP is diastolic blood pressure; mmHg is millimeters of mercury; mg/dl is milligrams per deciliter; HDL is high density lipoprotein; LDL is low density lipoprotein; cardiac functional capacity is rated on a 15-point scale; higher scores indicate better cardiac function. MBMI is the Benson-Henry Mind/Body Medical Institute. Ornish is The Dean Ornish Program for Reversing Heart Disease.

Selected results from the baseline survey are shown in Table 4. The overall response rate was 79%. Participants in the Ornish program were more self-confident of their ability to lose weight and to keep it off than were those in the MBMI program (p<0.001). Most participants indicated they were receiving very good or excellent support from friends or family, and more than half had smoked over 100 cigarettes at some time during their lifetimes. No patient was smoking at the time of enrollment, since both programs required smoking cessation as a condition for program eligibility.

**Table 4:** Participants’ Responses to Questions about Health-Related Habits and Attitudes.

		MeanValues		p-value ofdifference
Topic of Question	N	Ornish	MBMI	
I would achieve a 15 lb. weight loss in 3 monthsif I tried to.(1 = very likely; 5 = very unlikely)	329	2.1	2.8	0.001
If successful, I would keep weight off for 1 year (1 = verylikely; 5 = very unlikely)	332	1.9	2.3	0.001
Success in facing life’s challenges (3 questions on 4point scales and summed; 0-not at all true; 12 = exactly true)	461	6.9	6.8	0.53
Overall support from family and friends (1 = poor;5 = excellent)	459	4.4	4.4	0.59
My overall health is (1 = poor; 5 = excellent)	454	3.4	3.2	0.18
Ever smoked 100 cigarettes (% yes)	459	54.4%	57.4%	0.59

MBMI is the Benson-Henry Mind/Body Medical Institute. Ornish is The Dean Ornish Program for Reversing Heart Disease.

### Changes in Cardiac Risk Factor Levels

Mean changes in cardiac risk factor levels during the lifestyle programs are shown in Table 5. By the end of the intense 3-month intervention, statistically significant improvements had occurred in most risk factors in both Ornish and MBMI program participants. The exception is HDL cholesterol, which decreased in Ornish program participants. Cardiac functional capacity improved significantly in participants in both programs. Supplemental analysis found that changes in total cholesterol and LDL cholesterol were of similar magnitudes whether or not participants were receiving antilipemics at baseline (data not shown). The magnitudes of change in risk factors at each time point were almost always greater in participants who remained active in the program (FULL). However, the directions of changes, levels of statistical significance, and time patterns were similar in the ANY and FULL groups.

**Table 5:** Average Changes in Cardiac Risk Factor Levels from Baseline Values by Program and Time Point.

	Ornish Program (N = 140)						MBMI Program (N = 440)				
Risk Factor	3 mos. ANY n = 137	3 mos. FULL n = 64	12 mos. ANY n = 94	12 mos. FULL n = 64	24 mos. FULL n = 66	3 mos. ANY n = 431		3 mos. FULL n = 255	12 mos. ANY n = 317	12 mos. FULL n = 255	24 mos. FULL n = 255
BMI (kg/m3)	−1.3‡	−1.6‡	−1.8‡	−2.1‡	−1.3‡	−0.6‡		−0.7‡	−1.0‡	−0.9‡	−0.5‡
SBP (mmHg)	−4.0†	−6.3‡	−4.8*	−7.5†	−4.0N	−3.1‡		−4.0‡	−5.6‡	−6.4‡	−6.9‡
DBP (mmHg)	−2.2N	−3.4†	−3.4‡	−4.5‡	−1.2N	−3.1‡		−3.9‡	−4.3‡	−4.9‡	−3.5‡
Total cholesterol (mg/dl)	−19.1‡	−23.8‡	−9.4*	−11.4*	−12.6*	−8.9‡		−9.6‡	−8.4‡	−9.1‡	−8.2‡
LDL (mg/dl)	−10.9‡	−14.9‡	−4.9 N	−6.0N	−10.5*	−7.4‡		−7.6‡	−7.5‡	−7.9‡	−9.1‡
HDL (mg/dl)	−5.1‡	−5.8‡	−1.1 N	−1.6N	1.6N	0.8*		1.0*	2.9‡	3.2‡	2.7‡
Triglycerides (mg/dl)	−11.5N	−14.3N	−14.9*	−15.1N	−13.0N	−10.0‡		−10.5†	−17.0‡	−17.3‡	−8.4*
Cardiac Functional Capacity (METs)	1.2‡	1.4‡	1.5‡	1.6‡	1.1†	1.5‡		1.6‡	2.0‡	2.0‡	1.6‡

Statistical significance: N denotes not significant, *p<0.05, †p<0.01, ‡p<0.001.

MBMI is the Benson-Henry Mind/Body Medical Institute. Ornish is The Dean Ornish Program for Reversing Heart Disease. BMI is body mass index; SBP is systolic blood pressure; DBP is diastolic blood pressure; mmHg is millimeters mercury; HDL is high density lipoprotein; LDL is low density lipoprotein; METs are metabolic equivalents; mos. denotes months of follow-up; ANY denotes all participants at the follow-up time; FULL denotes participants with final (24 month) data; n denotes the number of participants in that column. Data are not shown for 24 mos. ANY, but the patients and results are very similar to those for 24 mos. FULL.

In both programs, the magnitudes of changes were generally greatest at the end of the 12-month active intervention phase, but favorable changes were maintained at 24 months in participants who continued in the lifestyle program. Both programs achieved significant weight loss, but the magnitude of changes was greater in the Ornish program. Reductions in DBP and SBP were greater in the MBMI program. Sustained reductions in LDL occurred in both programs. The levels of HDL cholesterol increased steadily in MBMI participants, while the initial decrease in HDL in Ornish program participants returned to baseline levels.

**Figure 1:**
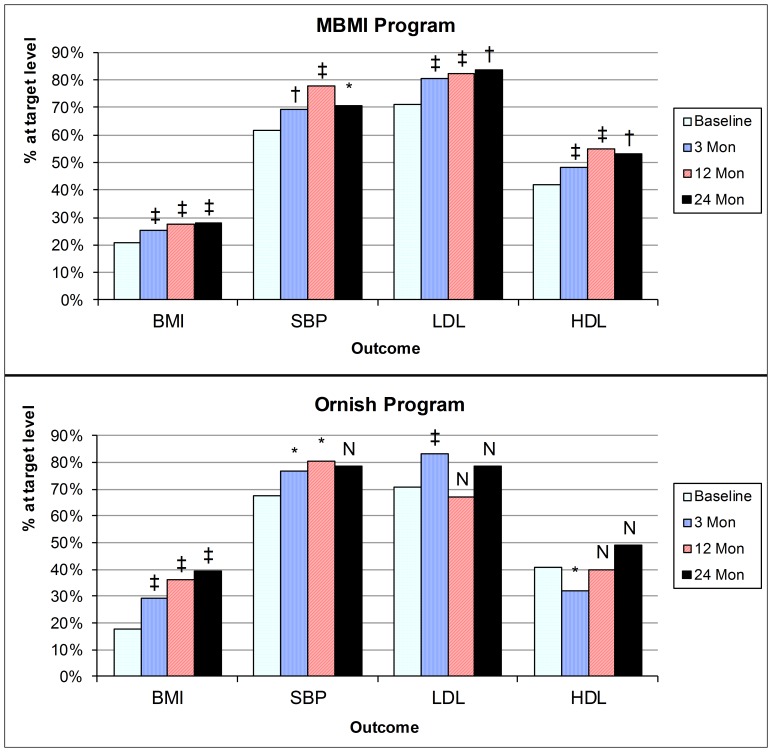
Proportions of Participants in Each Lifestyle Modification Program at Targeted Risk Factor Levels at Each Time Point. Notes: Targets defined as body mass index (BMI) < = 25; systolic blood pressure (SBP) <140 mm Hg; low density lipoprotein (LDL) <100 mg/dl; high density lipoprotein (HDL)>40 mg/dl (male) or>50 mg/dl (female). Mon denotes months. Statistical significance: * p<0.05, ** p<0.01, *** p<0.001, N denotes not significant. McNemar’s chi-square test was used for hypothesis testing. MBMI denotes the Cardiac Wellness Program of the Benson-Henry Mind Body Institute, Ornish is The Dean Ornish Program for Reversing Heart Disease.

The benefits of the programs are presented in terms of the attainment of therapeutic targets as defined by the American Heart Association in Fig. 1. These are the BMI of 25 or below, SBP of 140 mmHg or below, LDL of 100 mg/dl or less, and HDL above 40 mg/dl in men and 50 mg/dl in women. The proportions of participants with normal weights (BMI) increased from 19% at baseline to 40% after 2 years in the Ornish program and from 20% to 28% in the MBMI program. In both programs, more than 80% of goal weight achievement occurred during the first year, though some further progress was made in the second year for patients who remained active in the program. Maximum improvements in SBP control were achieved at the end of the active intervention year, with an increase from 61% to 78% in the MBMI program and from 68% to 80% in the Ornish program reaching SBP target levels. For LDL, 71% of each program’s participants were at goal levels at baseline. This proportion increased in the MBMI program to 82%, while changes in the Ornish program participants were less consistent. Increases in HDL occurred in the MBMI program, with results at two years being significantly higher than at baseline. In Ornish, HDL levels decreased during the first 3-months and then returned gradually to baseline levels.

It is likely that FULL participants (those with full data) were more motivated than those who missed one or more time points. Of the 32 possible comparisons (8 indicators×2 programs×2 time periods), 31 (97%) showed larger magnitude of changes for FULL participants compared to ANY at 3 and 12 months of follow up. This comparison suggests, as expected, that higher motivation was associated with greater change. On the other hand, even the participants who did not complete 24 month follow up achieved significant improvements at earlier time periods. Taking 3-month LDL changes as an example, the 62 Ornish and 115 MBMI dropouts after 3 months (i.e. those in ANY but not FULL) showed statistically significant reductions of 6.9 mg/dl (p<.05) and 7.0 mg/dl (p<.01), respectively.

Comparison of changes between the 12-month FULL and 24-month FULL participants shows the persistence of risk factor changes. Of the 16 possible comparisons (8 indicators times 2 groups), 13 show attenuation (though 9 still remain significantly better than baseline), while 3 show strengthening. Strikingly, LDL improvements were greater at 24 months than at 12 months for both programs. The lifestyle programs apparently contributed to careful attention to antilipemic medications and regular physical activity that are important for this risk factor.

### Risk Factor Changes Controlling for Participant and Program Characteristics

Multivariable regressions examine changes in cardiac risk factors adjusting for baseline patient-level characteristics including age, gender, race, type of qualifying clinical event, risk factor levels, program (Ornish or MBMI), and time period (Table 6). Decreases in risk factors were strongly associated with higher baseline values. These relationships may reflect greater incentives in participants with higher baseline values and/or regression to the mean. Participation in the Ornish program was associated with significantly greater reductions in BMI and SBP (controlling for a lower mean baseline SBP), favorable changes in LDL, and unfavorable changes (reductions) in HDL early during participation that waned over time. In patients who had received CABG surgery, BMI, SBP, DBP, and cardiac functional capacity each increased over the two-year period compared with participants who entered the lifestyle modification program with stable angina. These results probably reflect persistent motivation in patients who undergo cardiac revascularization. Participants who had had AMIs but did not have subsequent revascularization procedures gained weight during the lifestyle programs and increased their cardiac functional capacities. Women experienced greater increases in their HDL levels, lesser decreases in LDL levels, and less improvement in cardiac functional capacity than men, controlling for other factors. Significant improvements in BMI, DBP, and HDL persisted over the period of the demonstration, while those in LDL did not.

**Table 6:** Multivariable Relationships between Patient Characteristics, Type of Lifestyle Modification Program, and Changes in Cardiac Risk Factors over Two Years^a^.

IndependentVariable	ChangeinBMI	Sig.	SystolicBloodPressure	Sig.	DiastolicBloodPressure	Sig.	LDL	Sig.	HDL	Sig.	CardiacFunctionalCapacity(METS)	Sig.
Intercept	0.97		77.6	‡	56.7	‡	45.3	‡	12.4	†	6.6	‡
Baseline	−0.07	‡	−0.6	‡	−0.7	‡	−0.5	‡	−0.3	‡	−0.3	‡
Ornish program^b^	−0.61	‡	−3	†	0.9		0.3		−3.7	‡	0.1	
Quarter^c^	−0.26	‡	−0.8		−0.8	*	1.6	*	1.4	‡	0.3	‡
Quarter squared	0.03	‡	0.1		0.1	*	−0.2	*	−0.1	‡	−0.03	‡
Age (years)	0.004		0.04		0.2	*	−0.1		−0.01		−0.05	‡
Female	0.15		0.5		−1.2		6.8	‡	2.1	†	−0.6	‡
Non−white	0.07		−0.3		−0.01		−2.7		0.7		−0.2	
PCI^d^	0.19		0.9		1.1		−1.6		−0.95		0.3	
CABG^d^	0.67	‡	3.8	*	2.8	†	−2.1		−0.3		0.8	‡
AMI^d^	0.43	†	2.3		1.8		−2.7		0.3		0.7	‡

a Statistical significance (Sig.): *p<0.05, †p<0.01, ‡p<0.001.

b Reference group is the (MBMI) program.

c Quarter denotes quarter year (3-month period).

d Reference group is stable angina.

MBMI is the Benson-Henry Mind/Body Medical Institute. Ornish is The Dean Ornish Program for Reversing Heart Disease. BMI denotes body mass index; HDL denotes high density lipoprotein; LDL denotes low density lipoprotein; METS denotes metabolic equivalents; PCI is percutaneous coronary intervention; CABG is coronary artery bypass graft surgery; AMI is acute myocardial infarction.

**Figure 2:**
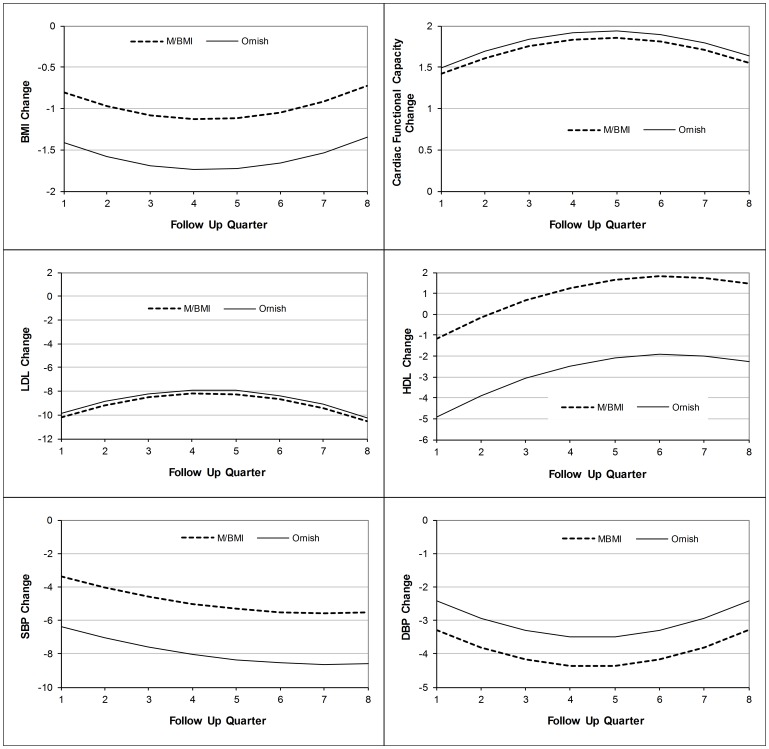
Risk Factor Changes in the Two Lifestyle Modification Programs Based on Multivariable Regressions with Quadratic Terms for Time. Notes: MBMI denotes the Cardiac Wellness Program of the Benson-Henry Mind Body Institute; Ornish is The Dean Ornish Program for Reversing Heart Disease. BMI denotes body mass index; LDL denotes low density lipoprotein; HDL denotes high density lipoprotein; SBP denotes systolic blood pressure; DBP denotes diastolic blood pressure.

Fig. 2 summarizes findings for risk factor changes in curvilinear relationships between risk factor changes and time. There was a gradual waning of favorable effects after one year for changes in BMI, LDL, DBP, and cardiac functional capacity in both programs, but sustained favorable effects for SBP and HDL levels.

### Clinical Events during the Intervention Period

Adverse clinical events during the active intervention period were reported by the clinical sites and evaluated by the Delmarva Foundation, an independent quality monitor. An adverse event was defined as one that resulted in a prolonged lapse in program participation and included any hospitalization, AMI, cardiac surgery or other revascularization procedure, cardiac catheterization or coronary angiography, emergency room visits, and death. [15] Overall, 24% of participants in the Ornish program and 16% in the MBMI program reported one or more adverse events. Death occurred in five participants (3.5%) in the Ornish program and three participants (0.7%) in the MBMI program (p = 0.026). The independent quality monitor thoroughly examined all deaths and concluded that none was related to participation in the lifestyle modification program.

## Discussion

Enrollment into Medicare’s Lifestyle Modification Demonstration was lower than anticipated by CMS despite an extension of the enrollment period to six years. Only 580 of the targeted 3,600 Medicare beneficiaries were enrolled. Reasons stemmed from stringent eligibility requirements, rigorous clinical and data protocols, the time demands of the programs on participants, and required participant copayments in some clinical centers. Enrollment was greater in the MBMI program than the Ornish program (440 vs. 140), possibly due to the Ornish program’s greater time demands, focus on adopting very low fat vegetarian diets, and enrollment by cohort at three or four month intervals rather than continuously. The adherence of enrollees to program requirements was excellent, with 98% completing the intense initial three-month period and 71% completing the year-long active program. Participants were highly motivated and may not be typical of all patients with coronary heart disease.

More than half of participants (56%) had had a recent coronary revascularization procedure as their qualifying events, while the remainder had either a recent AMI without revascularization or stable angina pectoris. This clinical spectrum is similar to findings in a study of Medicare beneficiaries who received traditional cardiac rehabilitation funded by Medicare. [16] All participants were under the active care of physicians at baseline, and nearly 90% were receiving antilipemic agents and 80% beta-blockers at the time of enrollment into the demonstration. Group means for total and LDL cholesterols were within normal ranges at the time of enrollment at 161 mg/dl and 89 mg/dl, respectively.

Statistically significant reductions were achieved by both programs in body weight, SBP, DBP, and LDL cholesterol, and were well-sustained in participants who remained in the program for two years. Changes in BMI were greater in Ornish program participants. With the Ornish program being more stringent and having lower enrollment, it may have attracted more highly motivated participants. While more highly motivated participants showed greater changes, even those with less motivation achieved significant improvements at 3 months. A transient reduction in HDL levels in Ornish program participants at the end of the three-month intensive intervention period returned to baseline levels after 12 months. This finding has been observed previously in individuals receiving a vegetarian diet. [17] Though lower levels of HDL are associated with a higher risk of coronary artery disease in epidemiologic studies, even after controlling for LDL levels, [18] the implications of these transient HDL changes are not known. The MBMI program achieved statistically significant increases in HDL levels at each time point.

Based on the number and description of adverse events, participation was felt to be safe. The rate of adverse cardiac events was consistent with the literature. For example, a review of PCI studies reported 868 adverse cardiac events in 6,922 patients over one year, a rate of 12.5% [19].

As no control group was available for this study, we searched the literature for relevant comparisons. We located a Canadian study with 126 participants in traditional CR [20] and a European trial with 68 patients with medical management but no CR or lifestyle program. [21] The data in both studies permitted calculation of risk factor changes from baseline to 3 months on subjects providing virtually all requested data, comparable to the FULL analyses in this study. In the study of traditional CR, three risk factors (SBP, DBP, and HDL) all showed significant improvements that exceeded those of the Ornish and MBMI programs. On the other hand, for the other three comparably measured risk factors (total cholesterol, LDL, and BMI), the gains were greater and statistically significant only in the Ornish and MBMI [20].

In the no lifestyle study, three risk factors (SBP, SBP, and BMI) showed significant improvements (declines) since baseline, one risk factor (HDL) showed a significant deterioration (increase) over baseline, while two risk factors (total cholesterol and LDL) showed no significant change. [21] Surprisingly, the pure controls achieved greater 3-month improvements in SBP and DBP than the lifestyle programs. This is probably because the pure controls had elevated average baseline blood pressures (SBP 150/DBP 92), whereas those of Ornish (129/72) and MBMI (135/73) participants were normal. Overall, the Ornish and MBMI programs generally achieved generally superior short-term results over these pure controls, with at least one of the lifestyle programs registering significant improvements on all indicators. These comparisons suggest that while lifestyle programs are better than no-lifestyle controls, the advantage of lifestyle programs over traditional CR may not lie in superior short-term results, but in documented sustained improvements through 24 months of follow up.

The clinical processes that lead to atherosclerosis may begin early in life and lead to cardiac events earlier or later in life. Effective primary or primordial prevention, including pharmacological treatment of risk factors in individuals with no overt evidence of cardiovascular disease, has been shown to reduce the frequency of later cardiac events and may be cost-effective or even cost-saving. [22] Primary prevention of cardiovascular disease has been strongly recommended by the Preventive Services Task Force based on a recent review of 74 clinical trials, [23], [24] which provided convincing evidence that improvements in total cholesterol, low-density lipoproteins, systolic and diastolic blood pressures, fasting glucose, diabetes incidence, and body weight can result from lifestyle counseling and treatment with medications in asymptomatic individuals with elevated risk factors. These findings underscore the importance of primary prevention and the risk factor benefits reported here in patients with symptomatic cardiovascular disease.

Strengths and limitations of our study need to be acknowledged. Major strengths lie in the systematic and complete data collected by the Delmarva Foundation on changes in cardiac risk factors and clinical events during the demonstration. Limitations relate principally to its observational pre-post design and the absence of a control group. Regression to the mean may play a role in explaining some of the observed favorable changes in cardiac risk factors. Also, findings in the highly motivated individuals who enrolled in the programs may not apply to less-strongly motivated individuals with CHD. Direct comparisons of outcomes with patients who received traditional cardiac rehabilitation or no rehabilitation will be required to determine the relative effectiveness and cost-effectiveness of these two types of programs.
